# Burn Injury-Specific Home Safety Assessment: A Cross-Sectional Study in Iran

**DOI:** 10.1371/journal.pone.0049412

**Published:** 2012-11-27

**Authors:** Shahnam Arshi, Homayoun Sadeghi Bazargani, Reza Mohammadi

**Affiliations:** 1 Department of Public Health Sciences, Shahid Beheshti University of Medical Sciences, Tehran, Iran; 2 Neuroscience Research Center, Department of Statistics & Epidemiology, Tabriz University of Medical Sciences, Tabriz, Iran; 3 Public Health Sciences Department, Karolinska Institute, Stockholm, Sweden; IUMSP, University Hospital Lausanne, Switzerland

## Abstract

**Background:**

The aim of this study was to assess the feasibility of injury specific home safety investigation and to examine the home safety status focused on burn related safety in a rural population in the North-West of Iran.

**Methods:**

A cross-sectional study was conducted on 265 rural households of rural Meshkinshahr, Iran. Cluster sampling method was used in 38 clusters with 7 households in each cluster. Clusters were selected on a probability proportional to size (PPS) basis using the available health census database called D-Tarh. Data were analyzed using the statistical software package STATA 8.

**Results:**

Possible risks were explored in fields of house structure; cooking and eating attitudes and behaviors; cooking appliances, specific appliances such as picnic gas burners, valors (traditional heaters), samovars (traditional water boilers), and air-heating appliances. Many safety concerns were explored needing to draw the attention of researchers and public health policy makers.

**Conclusion:**

Injury specific home safety surveys are useful and may provide useful information for safety promotion interventions.

## Introduction

The traditional view of injuries as being random events has resulted in a historical neglect of this area of public health [1]. Among the most challenging problems through this century will be to decrease the burden of injuries, which often results in either death or a considerable diminishment of quality of life among survivors. Much of this burden belongs to developing countries. Home, which is considered to be a safe haven by most people, can be a dangerous place where injuries frequently occur. This is especially true for particular groups of people such as children or the elderly. Although most adults are not aware of the risks within their houses, domestic injuries, particularly burns, are preventable threats to human health. Burns are considered a leading cause of morbidity and disability from injury in many low and middle income countries (LMICs). Even in high income countries, children and adults who spend the majority of their time at home, including mothers and the elderly, are at greater risk for some types of burns. In a previous study in rural areas of Ardabil Province in Iran, it was found that more than 90% of burns occurred at home [2]. A vital part of designing an injury prevention program is to map out the safety status at home regarding safety of the built environment, product safety and safety related behaviors of people. Contrary to the large number of hospital-based studies, population-based studies and home safety studies are limited in LMICs including Iran the sorely available epidemiological surveys have shown that 1% of all deaths in Iran may be due to unintentional burn injuries and the nonfatal burns requiring medical care are ported to have an incidence rate of 10.9 per 100,000 person-years while a recent population based study has reported an incidence rate of up to 340 per 100,000 person-years for minor and moderate burns [3]; [4]. National studies have shown that burns form the leading cause of domestic injuries among women of reproductive age in Iran comprising 43% of all domestic injuries in this group [5]. An abundant number of studies have been conducted worldwide to investigate home safety. Few, however, were conducted as target-group-specific investigations. Although there are studies on fall injuries that were conducted as an injury-specific assessment to measure the effect of a given interventional plan, we did not find previously published studies conducted as burn-specific home safety surveys. The aim of this study was to assess the feasibility of injury specific home safety investigation and to examine the home safety status focused on burn related safety in a rural population in the North-West of Iran. The home safety status was specifically assessed considering different aspects of appliances, house structure and household related safety.

## Methods

The study was conducted in Meshkinshahr District in Ardabil Province, North-West of Iran. Meshkinshahr district will be the area of intervention in the Ardabil study on burn epidemiology. A cross-sectional household survey was conducted over the period 2007–2008. A total of 265 rural households in Meshkinshahr District were enrolled. The sample size was estimated to cover variations of relative frequency from 0.15 to 0.85 with 5% precision and a 95% confidence level for categorical measures. This range covered assumed proportions of major categorical variables of interest including prevalence of common unsafe cooking behaviors, using faulty cooking appliances, using faulty air heaters, using faulty gas stoves and lacking a separate kitchen at house. Due to unavailability of previous reliable information for the main continuous measures, 95% confidence intervals were planned to be reported in order to make it possible to assess the precision of the point estimates. The cluster sampling method was used to draw samples in 38 clusters with a pre-specified cluster size of 7 households. Clusters were selected on a probability proportional to size (PPS) basis using the available health census database called D-Tarh. After determining the starting point to survey the households in each cluster, consecutive households were enrolled following the right-hand rule. Households established in Meshkinshahr District for at least six months and willing to participate were considered eligible. No institutional participants were enrolled.

A questionnaire for interview was developed, assessed and improved to be used as data collection tool in this survey. The main data being collected were on house structure and decoration; cooking and heating appliances; and people's knowledge, attitude and behaviors. The study units differed as houses, household members, cooking appliances, such as stoves, samovars etc.; heating appliances, dishes and potholders. A valor is a type of kerosene heater that is not connected to a chimney. It is used in some areas of Iran, especially the rural areas, for a dual purpose of cooking and heating the air.

A samovar is a heated metal container traditionally used to heat and boil water for making tea, mostly in Iran, Turkey, and Russia [2]; [6].

Several measurements were done regarding the safety related to each sampling unit. If more than one appliance existed in each household, the one which was used more frequently was assessed.

The interviewers were bachelor and master students along with district healthcare workers living in Meshkinshahr district. To minimize selection bias and information bias, several measures were taken. Data collection was performed at a time convenient to the household. The village healthcare worker accompanied the interview team to introduce them and explain the purpose of study. In consideration of cultural issues, interviewers were chosen tactfully and each team included a female and a male interviewer. Most of the interviews took place when both the housewife and household head, if not the same, were present. Interviewers were trained by the main researcher to do the data collection. A pilot survey was done in a test cluster before starting the main survey. To increase the validity of collected data, the main researcher checked the data from the first household interviewed by each team, routinely and later during the survey, in a random manner.

Data were analyzed using the statistical software package STATA 8. Descriptive statistics and graphs were produced. For normally distributed numeric measures, means were reported along with standard deviations (SD) or 95% confidence intervals (95% CI). In order to appropriately manage the design effect due to cluster sampling, Taylor-linearized variance estimation was used to calculate the confidence intervals. For those variables with skewed distribution, median values were reported along with interquartile ranges (IQR).

## Results

**Figure 1:**
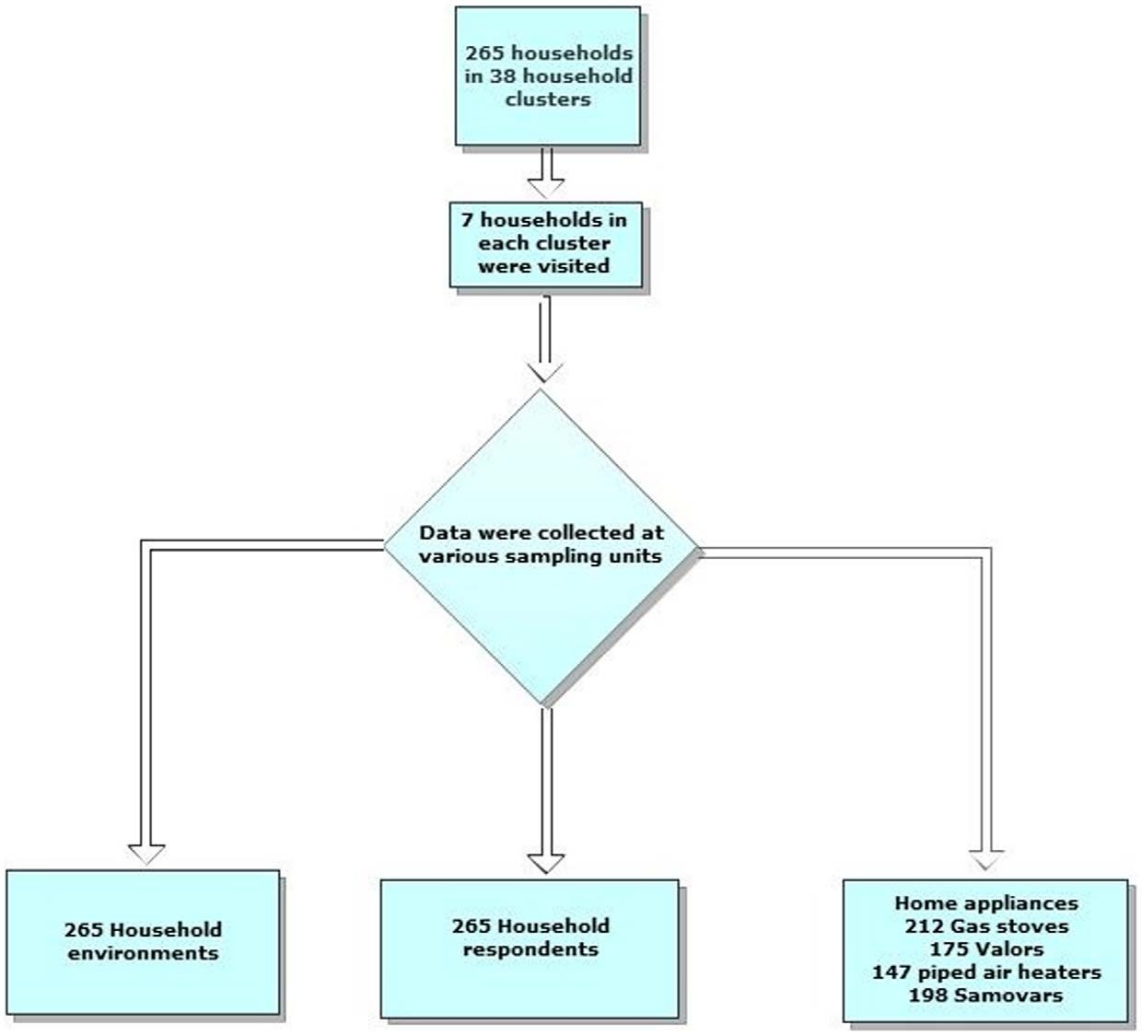
The sampling flow diagram.

265 households were enrolled. One household out of the 266 predefined sample size of households refused to participate in the study with no specific reason except for the fact that the father of the family was not at home(Fig. 1).

### House structure

The houses were made of loam lumps in an old fashion in 116 cases (44%). The mean roof area was 84.95 m^2^ (SD: 39.4).

The availability of different rooms is given in table 1. The smallest rooms were bathrooms and kitchens and the largest guest rooms.

**Table 1:** Availability of different rooms in houses.

Room name	No separate rooms available		One room available		Two rooms available		>2 rooms available		Total mean area (square meters)
	N	%	N	%	N	%	N	%	
Bedroom	75	28.3	147	55.5	39	14.7	4	1.5	23.4
Kitchen	105	39.6	159	60	1	0.4	0	0	11.4
Quest room	167	63	91	34.3	6	2.3	1	0.4	28
Living room	28	10.6	224	84.5	11	4.1	2	0.7	22.5
Bathroom	136	51.3	129	48.7	0	0	0	0	5.3

In 221 of 265 houses (84.4%) there was a height difference between the kitchen floor and kitchen entrance. The median height difference was seven centimeters with an interquartile range of six centimeters. Living rooms were furnished with chairs or armchairs only in three houses.

### Cooking

#### Cooking place and appliances

The kitchen was the exclusive place of cooking in 106 households (40%; 95%CI: 34.1–46.2%) and it was the usual place of cooking in 44 households (16.6%; 95% CI: 12.3–21.6%). The living room was the exclusive place of cooking in 32 households (12.1%: 95% CI: 8.4–16.6%) and the corridors in 19 households (7.2%; 95% CI: 4.4–11%). Living rooms were the usual place of cooking in five households and corridors were so in 21 households (7.9%; 95% CI: 5–11.9%).

Table 2 gives the frequency distribution of various types of cooking appliances used at home.

**Table 2:** Frequency of use of different types of cooking appliances.

	Always		Usually		Sometimes		Rarely		Never or not applicable	
	Number	%	Number	%	Number	%	Number	%	Number	%
Gas burner(multi burner)	75	28.3	63	23.7	11	4.1	13	4.9	103	38.8
Gas burner(single burner)	19	7.2	24	9.1	14	5.3	19	7.2	189	71.3
Picnic Gas burner	27	10.2	49	18.5	51	19.2	34	12.8	104	39.2
Valor	16	6	29	10.9	39	14.7	39	14.7	142	53.5
Firewood	3	1.1	12	4.5	12	4.5	30	11.3	208	78.5
Other	3	1.1	10	3.8	1	0.4	4	1.9	246	92.8

#### Gas stoves

The mean life span of gas stoves in use was seven years (SD: 6.1). The mean number of burners per gas stove was 2.9 (SD: 1.1). 136 (64%) of the 212 gas stoves had three burners, followed by five burners in 26 gas stoves (12.2%). The mean length of connecting gas tubes was 145 centimeters (95% CI: 124.1–165.9). These gas tubes were not replaced for a median of two years with an interquartile range of four years.

Only 14 (7%) gas stoves had thermocouples. Only nine gas stoves had built-in lighters. In 31 cases, gas stoves had broken legs. In 127 cases burners had safety adjusters for low flame. An earth safety wire was provided in 27 gas stoves.

The mean of the shortest distance between a flammable material and the nearest burner was 113.2 cm (SD: 88.7). In 25 of 212 (12%) households, gas burners were either rocking or placed unsafely.

About one-third of the gas stove repairs (7 out of 19 repairs) were done by people who lacked the technical competence such as family members, friends or neighbors.

A safety check on the gas burners in the last six-month period had been carried out in 70 households (26.4%, 95% CI: 20.2%–33.1%). Some consumer attitudes regarding gas-burning cooking appliances are given in table 3.

**Table 3:** Some user attitudes regarding gas burning cooking appliances.

		Completely agree	agree	No idea	Disagree	Totally disagree	Don't know about it
1	Gas stove needs to be checked periodically by an expert	43	136	21	10	5	
2	Domestic gas stoves need to be started by professionals	53	146	28	18	3	
3	Gas tubes connected to gas stoves need to be replaced after 2 years.	38	125	68	13	2	
4	It is important that your gas stove has a thermocouple	24	58	50	4	1	110
5	We can light matches to check for gas leak	30	120	17	32	24	27
6	A gas stove shouldn't be placed in wind flow direction	48	154	11	23	14	
7	The floor where we place a gas stove must be even	68	165	6	8	3	
8	The house wife needs to learn about how to use gas burning cooking appliances	66	145	26	10	1	

**Figure 2:**
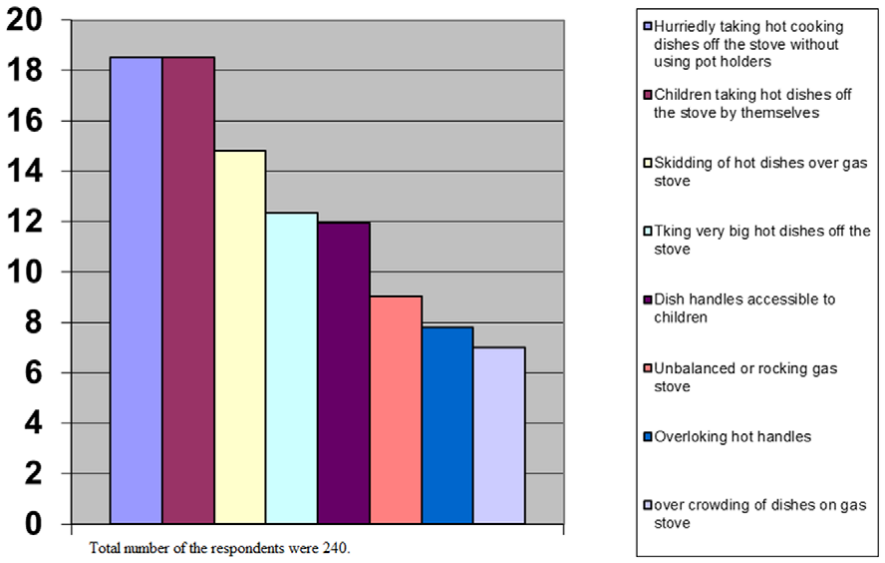
The answers of housewives to the question that what can be the most important cause of burn injuries while cooking on stove.

Answering to our question “How do you choose which burner on your gas stove has to be used for your usual cooking jobs?”, 30 respondents (12.5%; 95% CI: 8.6%–17.4%) declared their main criteria to be the cooking time and 80 (33.1%; 95% CI: 27.4%–39.7%) chose the burner based on compatibility of cooking dish size and burner holder size. Fifty-two (21.5%; 95% CI: 16.6%–27.4%) said they usually choose the burners that are easier to reach and comfortable to work with. Twenty (8.3%; 95% CI: 5.2%–12.6%) said they choose rear burners due to safety reasons. The respondents' views about the suitable time to replace the gas connection tube of the gas stove are presented in figure 2.

#### Picnic gas burners

The main alternative cooking appliance were picnic gas burners, in 164 households (61.9%; 95% CI: 55.7–67.8%) e.g. in case of problems with supplying gas canisters, in households without piped gas supply.. The second alternative in this regard were valors in 69 households (26.1%; 95% CI: 20.9–31.8%).

#### Valors

Only 17 of 175 (9.7%) valors had a national standards accreditation mark. In 20 cases the upper part of the valor was not screwed to the base. The upper holding part of the valor was unstable in 54 cases and it was slant in 44 cases. 28 valors had kerosene leakage through its fuel container. 16 valors had instable or broken legs. 29 valors had red flames indicating problematic fuel consumption. 69 of 175 (39.4%) households with valors used them in living rooms most of the time. 14 of households (8%) used valors in corridors most of the times. The valors were usually refueled by the head of the family in 25 households (14.3%; 95% CI: 9.5–20.4%), by the housewife in 126 households (72%; 95%CI: 64.7–78.5%), and by kids in 24 (13.7%) of households. In 46 households (28.4%; 95% CI: 21.6–36%) the last refilling of the valor was done without first extinguishing it.

#### Pot holders

Of the 224 households that owned either safety gloves or traditional pot holders for carrying hot dishes, only among 134 (60%; 95% CI: 53.1–66.3%) they were placed in a predefined location for easy reach.

In 42 (15.8%) households there was at least one occasion of not using pot holders during the last month before study while it was necessary to use them. The median frequency of occurrence of such risky behavior was twice per month with an interquartile range of one. The pot holder not being in easy reach was stated to be the reason for not using it in 50% of these cases (95% CI: 37.1%–62.9%). Ignoring the necessity to use pot holders and hurriedness were the other two main reasons for not using a pot holder when necessary. In 41% of the times people had used their sleeves instead of pot holders (95% CI: 28.2%–53.8%) and in 25.3% they relocated the hot dishes barehanded (95% CI: 13.7%–36.9%).

#### Samovars

A samovar was the main appliance used to boil water for tea in 198 of the households (75%; 95% CI: 69.3–80.1%). In 132 (66.5%) of those, these were kerosene samovars. The findings regarding samovars are published elsewhere [6].

### Air Heating

Only 23 (15.6%; 95% CI:9.8–20.4%) of the 147 Iranian kerosene air heaters had a national standards accreditation mark. Eighty-four (57.1%; 95% CI: 48.7–65.3%) lacked a fuel adjuster, 65 (44.5%; 95% CI: 36.3–53%)lacked a safety locker tap on fuel tank, and 29.8% (95%CI: 22.6–38%) lacked an outer heat shield. A heat protection shield existed between the fuel tank and burner wall in 107 kerosene heaters (73.8%; 95% CI: 65.8–80.1%) of kerosene heaters. In 75.1 percent a separate part was used to insulate the hot bottom of the heater from the underlying roof clothing. It was iron-made in 109 heaters (79.4%; 95% CI: 67.3–82%). A fuel leak was detected by the researchers in 11 heaters. Most of the airing holes of the burner were blocked in 29 heaters (21.2%; 95% CI: 14.6–28.8%).

Seven air heaters had an unstable placement. Nearly half of the respondents used to fix their heaters themselves when needed. The air heaters were misused for boiling water and cooking purposes in 105 cases (73.9%; 95% CI: 65.9–81%) cases. Utensils used for cooking or boiling on air heaters had unstable placement in 11 cases (7.8%; 95% CI: 4–13.5%).

### Knowledge and attitudes

**Figure 3:**
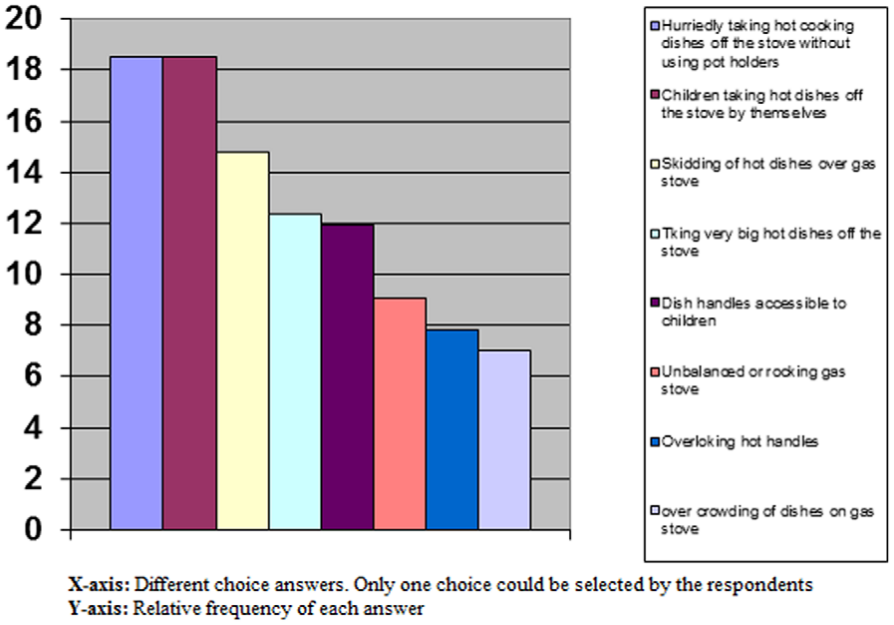
The answers of housewives to the question that what can be the most important cause of burn injuries while cooking on stove.

We asked parents at what age they think the boys and girls can help the adults in cooking or carrying hot food and tea. The mean age considered by the parents to be safe for the children to help in cooking was 10.7 (SD 2.5) years for girls and 13.3(SD 3.1) years for the boys. The mean age considered by the parents to be safe for children to help in serving tea or hot food was 9.6(SD 2.5) years for girls and 12.3(SD 3.1) years for boys. Housewives were asked to answer a picture question and choose one of 8 pictures which they thought was the safest situation of setting cooking dishes on a given 5-burner gas stove. Only 20 of 208 subjects (9.6%; 95%CI: 6–14.5%) chose the safest situation. Thirty-nine subjects (18.7%; 95% CI: 13.7–24.7%) chose a low risk situation, the remaining 149 chose higher risk settings. The risks overlooked by participants were as follows: letting the handles of pans to be accessible for children, using cooking dishes not compatible to the size of burners, using front burners while suitable back burners are free and using top samovars (samovar like kettles with taps that also hold pots on top) with their taps outwards accessible for children. The answers of housewives to the question “What can be the most important cause of burn injuries while cooking on stove?” are given in figure 3.

The mean knowledge score of the participants regarding precautions to be taken in case of gas leakage was 4.1 out of a maximum score of eight (SD: 2.1).

We showed the Iranian national standards accreditation mark both to the housewives and family heads. 59 of 230 (26%) housewives and 71 of 194 (37%) family heads knew what it was.

143 of 229 (62.5%) households who answered the question whether they had received any information regarding burn related cooking safety gave a negative answer. Sixty-three of the others who answered yes had received information from TV broadcast.

Only 52 households (20.2%; 95%: 15.4–25.6%) had received some information on what to do in case of fire accidents. This figure was 15.1 percent for learning about suffocation and explosion accidents and it was 14.6 percent in case of electricity related accidents.

### Other

Children had access to kerosene storage in 81 of 205 households consuming kerosene as their main fuel (39.5%; 95% CI:32.8–46.6%). In 61 of these households (29.9%; 95% CI: 23.7–36.7%) there were kerosene containers without a fixed lid. Teapots used were not intact in 65 (27%; 95% CI: 21.6–33.2%) of households. Wiring was declared to be done by expert electricians in 74 households (27.9%; 95% CI: 22.6–33.7%) and not done by them in 39 households(14.7%; 95% CI: 10.7–19.6%). Others were not sure or didn't know about that. In 33(12.4%; 95% CI: 8.7–17%) of households exposures were detected in electricity wiring due to corrupted insulation or cutoffs.

There were either epileptics or persons with musculoskeletal limb problems in 32 (12.1%; 95%CI: 8.4–16.6%) households. In five of these households, such people used to help in preparing tea.

Eleven households reported history of hot dishes on gas stoves to be turned over by children during the last year before the study, and 10 households reported it done by an adult during the same period of time. In 35 of 232 households the lifetime history of experiencing a gas leakage event from a gas stove was positive. It had happened more than once in 18 households.

## Discussion

The majority of scalds or burns at home occur during the use of heating-cooking appliances such as samovars, gas stoves, valors and picnic gas stoves. In the present study a wide range of possible burn related home safety measurements were studied mainly focused on cooking and heating. Many risks related to the home environment, cooking and heating appliances and safety-related behavior were explored in this setting.

It seems that, except for the fire burns, materials used in house structure may not have a major role in burn injuries. However, there is some evidence and logical plausibility that home structure, home size and internal architecture of houses may have a possible role in increasing the injury likelihood specially in case of fires, child burns and fall injuries [7]–[12]. A WHO report on child injury prevention considers absence of flame retardant household materials and lack of separation between cooking area and other areas as factors that increase the risk of injuries [9]. It is also stated that the structural design of urban homes may be a significant barrier to home safety-product usage [10].

Home structure may be considered the factor that is the least modifiable in injury prevention. It depends on socioeconomic level, cultural context and existing legislation. Legislation failures in addressing many hazards in housing and the need for new standards of assessing domestic conditions is being considered in some countries [13]. Very cost-effective interventions based on separation and isolation techniques for the prevention of injuries in Bangladesh and Norway were shown to be beneficial in preventing childhood burn injuries (add reference here). Such interventions have been made based on results of well-designed earlier population based studies [14]–[17].

Some of the explored risks in our study were related to cooking appliances. These included production safety, deterioration of cooking appliances over time and user behaviors.

Traditional kerosene and paraffin cookers, heaters, lamps and samovars have been shown to be of importance as a source of possible burn events. Moreover, based on geographical and cultural differences as well as resource availability, sometimes other types of appliances are used that may be the source of safety concerns. Kerosene pressure stoves mainly known as primuses, for instance, are produced in different sizes and forms in some countries [18]–[21]. sometimes other types of traditional kerosene appliances,such as Valors and Aladins in Iran, are to be considered for their risks and sometimes these can be paraffin cookers [2]; [6]; [7]; [22]–[26].

As found in this study, portable or picnic gas stoves turn out to be a popular cooking or even heating appliance for everyday home usage in some households. Studies stress the relevant risks either in product safety and design or with mishandling or improper use of them [2]; [7]; [12]; [27]–[29].

Microwave ovens were not included in our study because they were quite rarely used in rural areas of Iran. Nevertheless, there are several reports of burn injuries due to microwave ovens. However, design or product safety doesn't seem to be the problem in this regard and mainly the problem arises of improper use and unsafe behaviors [30]–[33].

Tandirs are specific baking appliances which are not a major problem of importance in Iran nowadays. some studies from Turkey state a major role for tandir burns that are severe most of the times [34]; [35].

Considering our findings and the large available evidence regarding cooking and heating appliances it seems quite essential for any home safety study to assess safety of such appliances and also investigate the related consumer behaviors.

Knowledge, attitude and behaviors are common factors assessed through some home safety studies. But there are also several interventional studies trying to assess the effect of education on home safety improvement.

Eichelberger et al in their study about child safety found that parents know little about dangers of pedestrian and bicycle injuries, burns, and drowning (add reference to Eichelberger et al.). Parents frequently mentioned “being careful” when describing precautions to reduce the risk of unintentional injury rather than mentioning known safety precautions. Parents of lower socioeconomic status demonstrated a more limited understanding of child safety [36]. Most of the previous home safety studies have considered measurements of knowledge, attitudes and practice(KAP) in their study but the assessments have mainly been focused on knowledge and attitude while less attention is paid on behaviors. Secondly, many aspects of burn related home safety have not been well addressed in previous research. This emphasizes the necessity of conducting problem oriented or burn-specific home safety surveys. Previous studies regarding home safety have usually been general or at most target-group-specific studies. Moreover, the available studies have not usually combined environmental assessments with the safety related behaviors. We found many burn related safety problems in our study population that may be similar in other rural areas.

### Limitations

The main limitation of this study was that it was conducted just in a rural population. Replication of this study or conducting similar studies in urban areas and various settings is recommended. However, this doesn't jeopardize the generalizability of the results to the study population and also the results of the study may reasonably be generalized to rural communities in most parts of Iran and to some extent to the southern rural turkey or northern rural Iraqi provinces. Moreover, it is methodological value can be beneficial to the international audience.

### Conclusion

Conducting a burn-specific home safety survey in a rural area of Iran revealed several areas of risk regarding burn injuries. However, future analytical studies are needed to consider them as real risk factors for burn injuries. Injury specific home safety studies are feasible and can provide rich information for safety promotion purposes.
