# Correction: Burn Injury-Specific Home Safety Assessment: A Cross-Sectional Study in Iran

**DOI:** 10.1371/annotation/52450b48-3eeb-4981-8c41-ff570f7298ad

**Published:** 2013-05-03

**Authors:** Shahnam Arshi, Homayoun Sadeghi-Bazargani, Reza Mohammadi

There was an error in the name of the second author.

The correct name is: Homayoun Sadeghi-Bazargani

The correct citation is: Arshi S, Sadeghi-Bazargani H, Mohammadi R (2012) Burn Injury-Specific Home Safety Assessment: A Cross-Sectional Study in Iran. PLoS ONE 7(11): e49412. doi:10.1371/journal.pone.0049412 

